# In vitro assessment of antitumor immune responses using tumor antigen proteins produced by transgenic silkworms

**DOI:** 10.1007/s10856-021-06526-6

**Published:** 2021-05-17

**Authors:** Kanae Yamada, Kei Masuda, Shota Ida, Hiroe Tada, Minori Bando, Kanako Abe, Ken-ichiro Tatematsu, Hideki Sezutsu, Tetsunari Oyama, Kazuaki Chikamatsu, Shigeki Takeda

**Affiliations:** 1 Faculty of Science and Technology, Division of Molecular Science, Gunma University, Kiryu, Gunma 376-8515 Japan; 2 Department of Pathology, Gunma University Graduate School of Medicine, Maebashi, Gunma 371-8511 Japan; 3 Department of Otolaryngology-Head and Neck Surgery, Gunma University Graduate School of Medicine, Maebashi, Gunma 371-8511 Japan; 4 Transgenic Silkworm Research Unit, Institute of Agrobiological Sciences, National Agriculture and Food Research Organization, Tsukuba, Ibaraki 305-8634 Japan

## Abstract

The evaluation of antitumor immune responses is essential for immune monitoring to predict clinical outcomes as well as treatment efficacies in cancer patients. In this study, we produced two tumor antigen (TA) proteins, melanoma antigen family A4 and wild type p53, using TG silkworm systems and evaluated anti-TA-specific immune responses by enzyme-linked immunosorbent spot assays in patients with head and neck cancer. Eleven (61.1%) of 18 patients showed significant IFN-γ production in response to at least one TA; however, the presence of TA-specific immune responses did not significantly contribute to better prognosis (overall survival, *p* = 0.1768; progression-free survival, *p* = 0.4507). Further studies will need to be performed on a larger scale to better assess the clinical significance of these systems. The production of multiple TA proteins may provide new avenues for the development of immunotherapeutic strategies to stimulate a potent and specific immune response against tumor cells as well as precise assessment of antitumor immune responses in cancer patients.

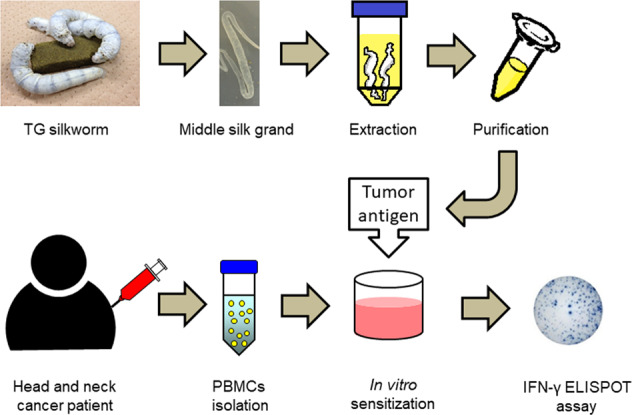

## Introduction

It is well known that immune status in cancer patients is systemically suppressed to some extent and that systemic immune activity predicts clinical outcomes [1–3]. Moreover, patients are treated with various immunosuppressive therapies, including chemotherapy and radiotherapy. Since cancer immunotherapy with immune checkpoint inhibitors (ICIs) has emerged as a promising treatment modality for cancer patients, there is increasing interest in precise evaluation of antitumor immune responses [4–6]. However, when patients with clinically diagnosed cancer have already progressed to the escape phase in the process of cancer immunoediting [7], antitumor immune responses can no longer control tumor growth. Nevertheless, the existence of antitumor immune responses, especially the acquired or adaptive immune response against tumor cells, implies better prognosis as well as treatment efficacy of cancer immunotherapy [8].

Currently, various blood-based immunoassays for detecting antitumor immune responses have been applied for immune monitoring in pre-clinical and clinical studies of cancer patients. Although several new approaches for immune monitoring, such as mass cytometry, mass spectrometry, and RNA-seq, are currently under development [9, 10], traditional immunoassays still have great potential to reliably and accurately measure the immune response of a patient against tumor cells [11, 12].

We previously reported the possible clinical application of a recombinant protein production system using transgenic (TG) silkworms [13]. Expression of proteins in silkworms was originally used for secretory proteins [14–16] and membrane receptors [17, 18], because the silk gland is a specific organ for secretory expression of silk fibroin. Moreover, the advantage of this secretory expression system is the supposed lack of contaminations by various kinds of substances that are expressed by the host cell or are present in the medium. Therefore, silkworms are expected to be suitable for the preparation of proteins acting on immune cells that are nonspecifically sensitive to trace amounts of contaminants. Additionally, since the recombinant protein expressed in the cocoon is expected be stored at room temperature (RT) without denaturation and degradation, the silkworm expression system could be applied for the production of vaccines and therapeutic proteins [19].

In the present study, two tumor antigens (TAs), melanoma antigen family A4 (MAGE-A4) and wild type p53 (WT-p53), were produced using TG silkworms. TA-specific immune responses in patients with head and neck cancer (HNC) were evaluated by enzyme-linked immunosorbent spot (ELISPOT) assays. Safe mass production of high-quality TA proteins from insects may provide new avenues for developing more effective and selective cancer immunotherapies.

## Materials and methods

### Generation of TG silkworms

All TG silkworm strains were generated and maintained at the Transgenic Silkworm Research Unit at the National Institute of Agrobiological Sciences (Ibaraki, Japan) as described previously [13]. Briefly, the silkworm strain w1-pnd, a non-diapausing strain that produces non-pigmented eyes and eggs, was used. The gene transfer vector carrying the AmCyan gene under a 3×P3 neuron-specific promoter was used. The human WT-p53 gene was kindly donated by Dr. Akarawa (Division of Cancer Biology, National Cancer Center Research Institute, Japan). The MAGE-A4 gene was prepared by PCR using a Flexi ORF Clone pF1KB9825 plasmid (Kazusa DNA Research Institute, Chiba, Japan) as a template. We inserted the target gene (human MAGE-A4 or WT-p53 gene fused with a signal peptide sequence at the start and a 6 x histidine tag sequence at the end) under yeast UAS of the transfer vector and named as pBac[UAS_MAGE-A/3×P3-AmCyan] or pBac[UAS_p53/3×P3-AmCyan]. One of the transfer vectors was injected into embryos at the pre-blastoderm stage with a helper plasmid for expression of a piggyBac transposase gene. G0 TG moths were mated with each other. G1 silkworms were screened using AmCyan expression in the embryonic compound eyes driven by a 3×P3 neuron-specific promoter at the late embryonic stage. For target gene expression, TG silkworm lines were then mated with the yeast Gal4-expressing strain carrying a GAL4 gene under a middle silk gland (MSG)-specific sericin1 promoter and a 3×P3-DsRed2 marker gene. F1 embryos expressing human MAGE-A4 or WT-p53 were screened based on fluorescence of AmCyan and DsRed2 using fluorescence microscopy (Olympus Corporation, Tokyo, Japan). The selected silkworms were reared on an artificial diet (Nosan Corporation, Yokohama, Japan) at 25 °C.

### Detection of expressed MAGE-A4 and WT-p53 in silkworm silk gland

Isolated MSGs from TG silkworms of the 5th instar were soaked in 1 mL of 20 mM phosphate (pH 7.2). Protein extracts where obtained after a few rounds of freeze-thawing. Protein supernatant (10 µg) was analyzed by SDS-PAGE using 4–12% gradient gels (NuPAGE Bis-Tris Gels; Thermo Fisher Scientific, Inc., Waltham, MA, USA). After electrophoresis, the gel was stained with 0.2% Coomassie Brilliant Blue R-250 (Nacalai Tesque, Kyoto, Japan). For western blotting, the gel was transferred onto PVDF membranes (Hybond P, GE Healthcare Life Sciences, Chalfont, UK), and incubated in blocking buffer (Blocking One, Nacalai Tesque, Kyoto, Japan) for 1 h at RT. We incubated the blotted membrane with a 1/1000 diluted anti-histidine tag primary antibody (A190–114A, Bethyl Laboratories, Montgomery, TX, USA) at 4 °C overnight. Subsequently, the membrane was washed three times with PBS with Tween-20 (PBST) [8 mM Na_2_HPO_4_, 2 mM KH_2_PO_4_ (pH 7.4), 150 mM NaCl, 3 mM KCl, 0.05% Tween-20] and incubated with a 1/20,000 diluted anti-rabbit IgG secondary antibody labeled with horseradish peroxidase (NA934, GE Healthcare Life Sciences) for 1 h at RT. Finally, the membrane was washed two times with PBST and immune-reactive protein bands were detected using ECL Prime reagent (GE Healthcare Life Sciences). An LAS-3000 Image Analyzer (Fujifilm Image Reader LAS-3000, version 2; Fujifilm Corporation, Tokyo, Japan) was used for visualization.

### Extraction and purification of recombinant MAGE-A4 and WT-p53

We dissected TG larvae on the 5th instar and isolated pairs of MSGs (~300 µg). Each pair of MSG was soaked in 20 mM phosphoric acid (pH 7.2) and gently shaken at 4 °C for 2 h. Extraction of target protein (MAGE-A4 or WT-p53) and fibril formation of fibroin were performed by a few rounds of freeze-thawing. Debris and fibers were removed from the extract by centrifugation (2300 × *g* for 10 min at 4 °C) and filtration (0.45 mm filter). The resulting protein extracts from MSGs were dialyzed against 20 mM phosphate (pH 7.4) (overnight at 4 °C). We loaded extracts onto a nickel affinity column (5 mL; GE Healthcare Life Sciences) equilibrated with 20 mM phosphate (pH 7.4) for purification of recombinant proteins, because the histidine tag was fused with the target proteins at the C-terminal end. After sample loading, the column was washed with 20 mM phosphate (pH 7.4), 20 mM imidazole, 500 mM NaCl, and recombinant MAGE-A4 or p53 was eluted with a solution containing 20 mM phosphate (pH 7.4), 500 mM imidazole, and 500 mM NaCl. Each fraction from the column was evaluated using 12.5% SDS-PAGE.

### Endotoxin assay

The quantification of endotoxin in the protein specimens were performed according to the Japanese Pharmacopoeia, 17th Edition. This endotoxin test listed is a method for quantifying endotoxins using a lysate reagent prepared from blood cell extracts of horseshoe crab. An endotoxin quantitative kinetic assay was performed using the Limulus Color KY (Wako, Japan) according to manufacturer instructions. A sample was mixed with the Limulus Amebocyte lysate reagent in the Limulus Color KY into a 96-well plate and placed in an incubating plate reader and absorbance was measured at 405 nm, 37 °C.

### Patient samples

Peripheral blood samples were obtained from 18 patients with HNC, and peripheral blood mononuclear cells (PBMCs) were isolated by density gradient centrifugation and cryopreserved until use. Patients did not receive any anticancer drugs, radiotherapy, or surgery before blood collection. Several clinical factors, including age, sex, primary tumor, *T* status, *N* status, *M* status, and stage, were evaluated. Patient characteristics are summarized in Table 1. This study was approved by the Institutional Review Board of Gunma University (HS2017-152) and written informed consent was obtained from each patient.

**Table 1:** Patient characteristics

pt-No	Age/sex	Primary	*T*	*N*	*M*	Stage	Tumor antigen expression	
							MAGE-A4	p53
1	68 M	Larynx	2	0	0	II	+	−
2	56 M	Hypopharynx	1	3	1	IVC	−	+
3	32 M	Oral cavity	4a	2c	0	IVA	−	−
4	62 M	Hypopharynx	2	2c	0	IVA	+	−
5	63 M	Oropharynx	4a	2b	0	IVA	−	+
6	71 F	Hypopharynx	3	2c	0	IVA	−	+
7	48 M	Oral cavity	4a	2c	0	IVA	+	+
8	65 M	Mucosal melanoma (Nasal cavity)	4a	0	0	IVA	NA	NA
9	58 F	Hypopharynx	3	2b	0	IVA	+	+
10	89 M	Larynx	2	0	0	II	+	−
11	68 M	Oropharynx	2	1	0	III	+	+
12	75 M	Hypopharynx	2	2b	0	IV	+	−
13	72 M	Larynx	2	0	0	II	+	+
14	72 M	Larynx	3	2c	0	IVA	+	+
15	70 M	Hypopharynx	3	2c	0	IVA	+	+
16	65 M	Larynx	3	2c	0	IVA	+	−
17	65 M	Hypopharynx	2	1	0	III	+	+
18	57 M	Hypopharynx	4a	2b	0	IVA	+	+

*NA* not available

### Immunohistochemistry (IHC) and evaluation

The tumor specimens from one of the patients were not available. Therefore, immunohistochemical analysis was performed in 17 HNC patients. The formalin-fixed paraffin-embedded surgical or biopsy specimens were cut into 3 µm sections and deparaffinized in xylene. Endogenous peroxidase activity in the sections was then blocked by 30 min incubation in 0.3% hydrogen peroxide in methanol. Antigen retrieval was achieved by boiling the samples at 98 °C for 30 min in citrate buffer (pH 6.0) or 98 °C for 30 min in 20% zinc sulfate solution for MAGE-A4 and p53 staining, followed by blocking using 1% bovine serum albumin containing 0.25% casein for 30 min. Slides were incubated at RT for 2 h with primary antibodies (anti-MAGE-A4 antibody [1:2000], clone 57B, MERCK; anti-p53 antibody [1:50], NCL-p53-DO7, NOVOCASTRA), and then incubated overnight at 4 °C. Subsequently, slides were incubated with secondary antibody (Histofine simple stain MAX-PO (MULTI), Nichirei). The reaction products were detected with 3,3′-diaminobenzidine (DAB, DOJINDO, Kumamoto, Japan) and counterstained with Mayer’s hematoxylin.

Slides were assessed by two independent investigators (S.I. and K.C.) in a blinded fashion using a Zeiss Axioscope light microscope (Carl Zeiss Microscopy GmbH, Jena, Germany). For MAGE-A4, each specimen was positive if specific staining was present. For p53, a microscopic examination of nuclear staining was performed and specific staining in >10% of the tumor cells was defined as positive expression as described in a previous report [20].

### In vitro sensitization and IFN-γ ELISPOT assays

PBMCs were thawed, washed, and cultured in the presence of recombinant protein (10 µg/mL of MAGE-A4 or WT-p53) in a final volume of 1 mL AIM-V medium supplemented with 10 IU/mL IL-2 and 5 ng/mL IL-7 in a 24-well tissue culture plate. After 4 days, 1 mL of AIM-V medium containing 10 IU/mL of IL-2 was added to each well. Following 3 days of incubation, PBMCs were harvested, washed, and tested using IFN-γ ELISPOT assays.

ELISPOT assays were performed using Human IFN-γ single-color ELISPOT kit (Cellular Technology Ltd., Cleveland, OH, USA) according to manufacturer protocol. Briefly, effector cells (1–5 × 10^4^ cells/well) were co-cultured with PBMCs (1 × 10^5^ cells/well) in the presence of MAGE-A4 or WT-p53 protein (10 μg/mL each) in a 96-well plate precoated with IFN-γ capture antibody. PBMCs stimulated with 10 μg/mL phytohaemagglutinin (PHA) were used as a positive control. The plates were incubated at 37 °C for 24 h. After incubation, the plates were washed and developed with anti-human IFN-γ (biotinylated) and streptavidin-alkaline phosphatase. The number of spot-forming-cells (SFC) in each well was automatically counted with a CTL-ImmunoSpot Analyzer (Cellular Technology Ltd.). The mean number of spots in control wells (no protein) was subtracted from the mean number of spots in experimental wells and the results are expressed as SFC per 5 × 10^4^ cells.

### Statistical analysis

GraphPad Prism version 8.0 (GraphPad Software, San Diego, CA, USA) was used to perform statistical analyses. Student’s two-tailed *t* test was performed to determine whether there was a significant difference between the number of SFC in protein-stimulated and unstimulated wells, as described by Nagorsen et al. [21]. The Fisher’s exact test of independence was used to determine differences in categorical variables. Kaplan–Meier curves were plotted and compared using the log-rank tests to compare survival curves between patients with and without TA-specific immune responses. *P* values < 0.05 were considered significant.

## Results

### Generation of TG silkworms expressing MAGE-A4 and p53 and preparation of tumor antigens

**Fig. 1:**
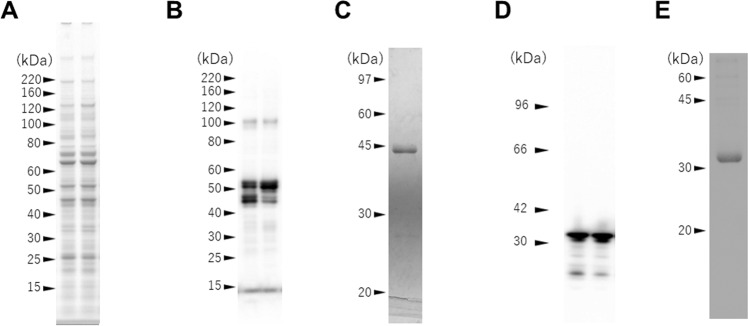
Expression of WT-p53 and MAGE-A4 in MSG of transgenic silkworms and its purification. **A** Protein extracts from the MSG of WT-p53 expressing TG silkworms were separated by SDS-PAGE using 4–12% gradient gel. **B** Western blot analysis of the MSG protein extracts for p53 with an anti-His tag antibody and 4–12% gradient gel. The two lanes correspond to the individual TG silkworm lines. **C** 12.5% SDS-PAGE analysis of the purified recombinant WT-p53. The MSG protein extracts were purified by nickel affinity chromatography and confirmed to be a single band at 44 kDa. **D** Western blot analysis of the MSG protein extracts for MAGE-A4 with an anti-His tag antibody and 8% polyacrylamide gel. The two lanes corresponded to the individual TG silkworm lines. **E** 10% SDS-PAGE analysis of the purified recombinant MAGE-A4. The MSG protein extracts was purified by nickel affinity chromatography and confirmed to be a single band at 36 kDa. Numbers on the left of each image indicate molecular masses (kDa)

We previously reported successful secretory expression and purification of MAGE-A4 [13]. The expression and purification of WT-p53 conjugated with a his-tag at the C-terminal end was performed in the same manner. At first, we analyzed the expression of recombinant WT-p53 in TG silkworm MSGs by SDS-PAGE (Fig. 1A) and western blot (Fig. 1B). A few immuno-reactive bands were observed in MSG extracts prepared from all TG lines. Next, we purified the recombinant WT-p53 using nickel affinity chromatography. The purified recombinant WT-p53 showed a 44-kDa single band on 12.5% SDS-PAGE (Fig. 1C). Since the calculated molecular weight based on the amino acid sequence consisting of 393 amino acid residues with a histidine tag was 44.5 kDa, we considered that the purified WT-p53 was not degraded. Recombinant MAGE-A4 was purified from TG silkworm MSGs and confirmed by nickel affinity chromatography and SDS-PAGE analysis as described previously (Fig. 1D, E) [13]. Although the purified yield of p53 (~40 µg per larva) was lower than that of MAGE-A4 (~170 µg per larva), a sufficient amount of antigen was prepared for the subsequent measurement of immune activity. Since a large-scale breeding method has already been established for silkworms, a mass production strategy for cancer antigens utilizing the silkworm expression system was almost ready. Finally, we tried to confirm that the purified MAGE-A4 and WT-p53 did not contain endotoxins that nonspecifically activate immune cells. The detection sensitivity limit of endotoxin was 0.001 units/mL; the detected amount of endotoxin in the purified cancer antigens was 0.002 units/mL, indicating that the antigens expressed in silkworm do not contain endotoxins.

### IHC for MAGE-A4 and p53 in HNC

**Fig. 2:**
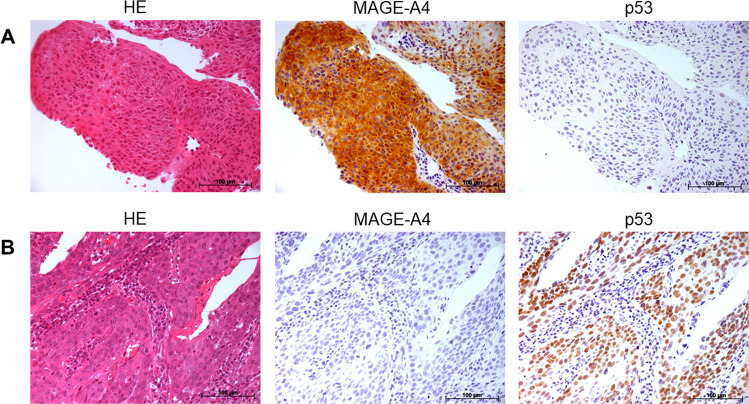
Representative hematoxylin and eosin (HE) staining and immunohistochemistry for MAGE-A4 and p53. **A** A MAGE-A4-positive and p53-negative case (patient no. 1). **B** A MAGE-A4-negative and p53-positive case (patient no. 6) (x200 magnification)

Immunohistochemical analysis was performed in tumor specimens obtained from 17 patients with HNC. Figure 2 shows the representative immunohistochemical staining of MAGE-A4 and p53. MAGE-A4 and p53 expression was detected in 13 (76.5%) and 11 (64.7%) patients, respectively (Table 1).

### Tumor antigen-specific IFN-γ production in patients with HNC

**Fig. 3:**
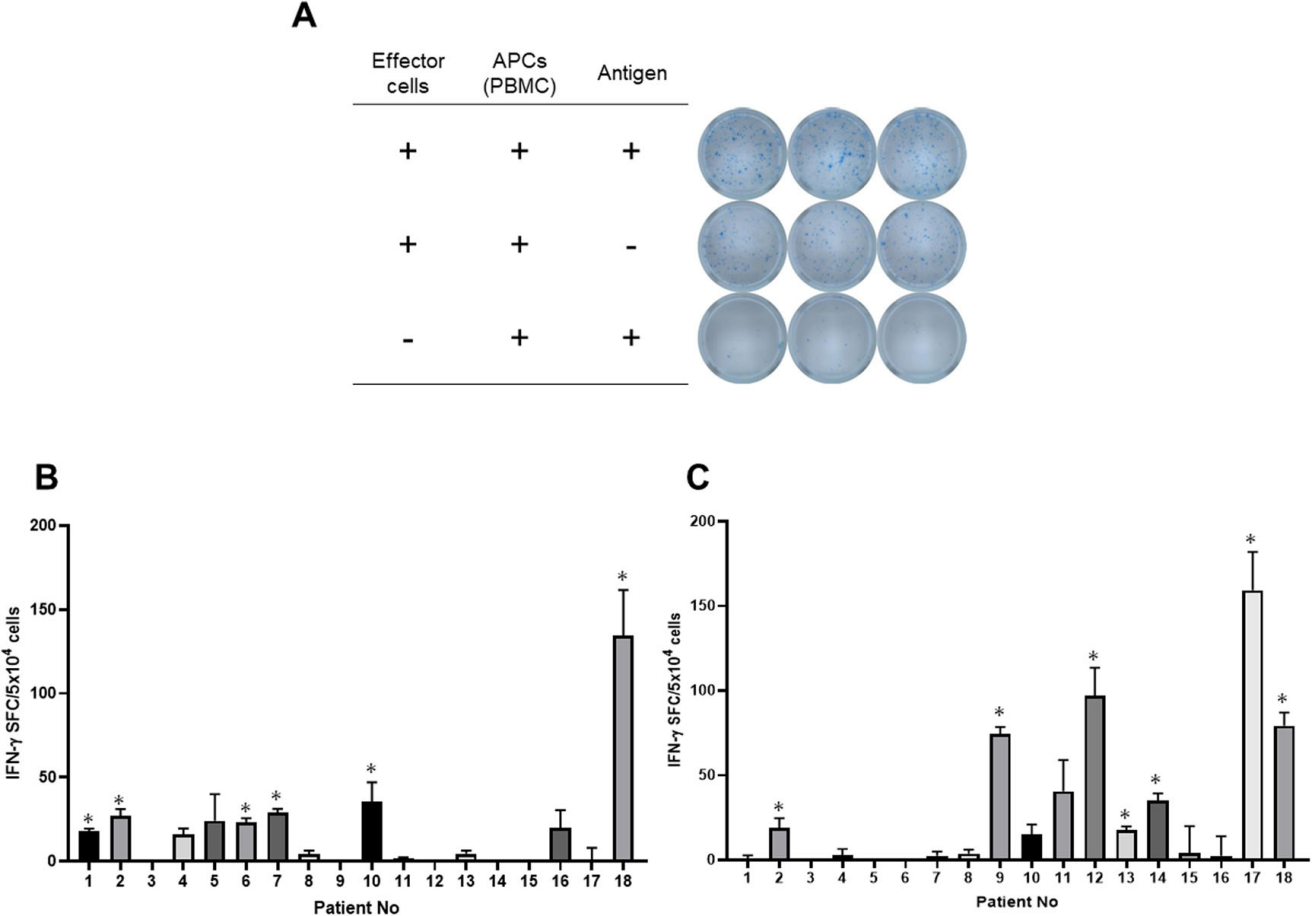
IFN-γ production in response to MAGE-A4 and p53 proteins produced by TG silkworm in patients with head and neck cancer. **A** Representative well imaging of ELISPOT assays detecting p53-specific T-cell response in pt-9. Quantification of the results from the ELISPOT assay detecting MAGE-A4 protein (**B**) and p53 proteins (**C**). The mean number of spots in the control wells (no protein) were subtracted from the mean number of spots in experimental wells. SFC, spot-forming-cells; *p < 0.05 compared to without protein, by Student’s two-tailed *t* test

PBMCs from 18 patients with HNC were stimulated with MAGE-A4 or WT-p53 protein and evaluated for TA-specific IFN-γ production (Fig. 3A). Eleven (61.1%) of 18 patients showed significant IFN-γ production in response to at least one of the TAs. Six patients (33.3%) responded to MAGE-A4 (range, 18–134 spots/5 × 10^4^ cells; Fig. 3B), whereas seven patients (38.9%) responded to WT-p53 (range, 18–159 spots/5 × 10^4^ cells; Fig. 3C). Among the six patients with MAGE-A4-specific responses, four (66.7%) had MAGE-A4-positive tumors. Regarding p53, six (85.7%) of the seven patients with WT-p53-specific responses showed p53 overexpression.

### Correlations between TA-specific immune responses and clinical factors

**Fig. 4:**
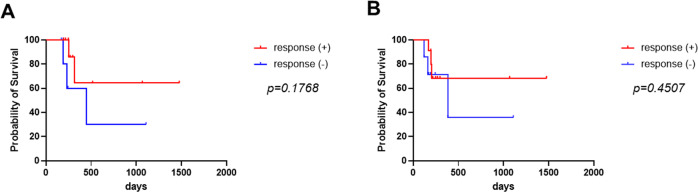
Kaplan–Meier curve and Log-Rank test for overall survival (**A**) and progression-free survival (**B**) for the comparison between patients with and without TA-specific immune responses

Finally, although the sample size was small, we investigated whether the patients harboring TA-specific immune responses are associated with clinical factors. Unfortunately, there was no significant correlation between TA-specific immune responses and any clinical factor including age, T classification, N classification, and stage (Supplementary Table 1). To evaluate the prognostic significance of TA-specific immune responses, Kaplan–Meier survival analyses were also performed between the two groups (Fig. 4). The patients with TA-specific immune responses appear to have a better prognosis for overall survival; however, there was no significant difference (overall survival, *p* = 0.1768; progression-free survival, *p* = 0.4507).

## Discussion

In this study, we reported the successful expression of WT-p53 in TG silkworms, following from the previous report on the expression of the cancer antigen MAGE-A4 [13]. Peptide antigens are generally employed to measure antitumor immune responses. Several reports are available for peptide antigens that exactly correspond to the epitope [22, 23]. In other words, information about the epitope sequence and MHC restriction are necessary for peptide antigen synthesis. In contrast, epitope information is not necessary for a full-length protein antigen. Currently, it is easy to prepare a full-length recombinant protein for an antigen. However, recombinant protein antigens often contain trace amounts of endotoxins derived from the expressing cells and medium, which nonspecifically stimulates immune cells and is an obstacle to measuring antitumor immune responses. In the TG silkworm expression system, recombinant proteins are secreted and expressed in the silk gland, which is completely sterile. We have shown herewith that the TG silkworm expression system prevents endotoxin contamination during the purification process. With the potential for large-scale breeding and ease for scale-up to industrial production of silkworms [15], the ability to obtain endotoxin-free recombinant antigens is a major advantage of our technique.

Antitumor immune responses mediated by T cells, especially cytotoxic T lymphocytes (CTLs), play crucial roles in tumor eradication; therefore, cancer patients with specific and potent T-cell responses against tumor cells most likely have better prognosis [2]. For assessment of TA-specific immune responses, several factors such as type, quantity, and quality of TAs should be considered. We previously demonstrated the production of a TA, MAGE-A4 protein, using a TG silkworm system as well as the induction of MAGE-A4-specific T cells from PBMCs of healthy donors [13]. In the present study, another TA, WT-p53 protein, was successfully produced using the TG silkworm system and used for the detection of TA-specific T-cell responses in patients with HNC. Both TAs, MAGE-A4 and p53, are broadly expressed in various kind of tumors. For instance, the frequency of MAGE-A4 expression was observed in 50% of head and neck squamous cell carcinomas, 24% of non-small-cell lung cancers, and 60% of esophageal cancers [24]. Moreover, as MAGE family members share a well conserved MAGE homology domain, which on average is 46% conserved amongst all human MAGEs, other MAGE family-derived antigens may also correspond with MAGE-A4 protein [25]. Similarly, it is widely acknowledged that TP53 is the most commonly mutated tumor suppressor gene in human cancer [26]. In the present study, 16 (94.1%) of the 17 tested HNC patients showed positivity for either MAGE-A4 or p53. Thus, the combined use of TA proteins could be applied to the assessment of antitumor immune responses in more cancer patients.

One of the most crucial biological factors for predicting clinical outcome is whether TA-specific adaptive immune responses are induced in cancer patients. The induction of strong TA-specific T-cell responses was shown to be dependent on the dose of TA, TA presentation by antigen-presenting cells, and TA-specific T-cell priming and activation. In the current study, 4 (30.8%) of the 13 patients with MAGE-A4-positive tumors and 6 (54.5%) of the 11 patients with p53-overexpressed tumors showed significant IFN-γ production, suggesting that TA-specific T-cell responses are induced in these patients. Both CD4+ and CD8+ T cells are capable of IFN-γ production; however the distribution of CD4+ and CD8+ T-cell responses against MAGE-A family antigens or p53 seems to vary with type of malignancy, type of antigen, and the level of antigen expression on tumor cells [27–29]. Exact determination of the actual frequency of CD4+ and CD8+ T-cell responses would require flow cytometric analysis of intracellular cytokines or ELISPOT assay with purified CD4+ and CD8+ T cells. Meanwhile, IFN-γ-producing T-cell responses against p53 seems to be stronger than those against MAGE-A4, which may be due to immunogenicity and the number of antigenic epitopes. Actually, Cheever et al. reported a prioritized list of cancer vaccine antigens based on predefined and pre-weighted objective criteria, and WT-p53 was ranked ninth of 75 representative tumor-associated antigens [30]. The patients with TA-specific immune responses appear to have a better prognosis for overall survival; however, there was no significant difference. Further studies using a larger patient population for validation of the clinical utility of TA proteins produced by TG silkworms are needed.

Recently, the emergence of ICIs has revolutionized the treatment of a variety of cancers, including melanoma [31], lung cancer [32], and HNC [33], and the biomarkers for therapeutic efficacy of ICIs have been extensively investigated [34]. Since the mechanism of action of ICIs reinvigorate tumor-reactive T cells by interrupting inhibitory signals of T-cell activation, ELISPOT assays using multiple TA proteins produced by TG silkworms would be an easy, fast, and reproducible tool for the evaluation of antitumor immune responses mediated by T cells in cancer patients treated with ICIs. There are currently ongoing studies that are assessing whether this assay provides sufficient value to predict the efficacy of ICIs treatment. Furthermore, the extraction of TA proteins at the mass production quality level may enable the clinical utility for cancer vaccines, which are another type of cancer immunotherapy.

## Conclusions

We have successfully produced two TAs, MAGE-A4 and p53 proteins, using the TG silkworm system and evaluated anti-TA-specific immune responses by ELISPOT assay in HNC patients. Eleven of 18 patients showed significant IFN-γ production in response to at least one TA. The production of such TA proteins may provide new avenues for the development of immunotherapeutic strategies to stimulate a potent and specific immune response against tumor cells, in addition to the precise assessment of antitumor immune responses in cancer patients. Moreover, it is expected that more detailed information on the immune status of patients will be obtained by preparing a wider variety of cancer antigens using TG silkworms.

## Supplementary information

Supplementary Table 1
